# Vitamin D Sufficiency of Canadian Children Did Not Improve Following the 2010 Revision of the Dietary Guidelines That Recommend Higher Intake of Vitamin D: An Analysis of the Canadian Health Measures Survey

**DOI:** 10.3390/nu9090945

**Published:** 2017-08-28

**Authors:** Lalani L. Munasinghe, Noreen D. Willows, Yan Yuan, John Paul Ekwaru, Paul J. Veugelers

**Affiliations:** 1 School of Public Health, University of Alberta, Population Health Intervention Research Unit, 3-50 University Terrace, 8303 112 Street, Edmonton, AB T6G2T4, Canada; lalani.m@ualberta.ca (L.L.M.); yan.yuan@ualberta.ca (Y.Y.); ekwaru@ualberta.ca (J.P.E.); 2 Agricultural, Food & Nutritional Science, University of Alberta, Edmonton, AB T6G1C9, Canada; noreen.willows@ualberta.ca

**Keywords:** vitamin D, diet, supplementation, children, 25(OH)D, dietary reference intake, sufficiency, Canadian Health Measure Survey, determinants, Canada

## Abstract

In 2010, the dietary guidelines for vitamin D for Canadians and Americans aged 1–70 years were revised upward. It is unknown whether the vitamin D status of Canadian children improved after 2010. We compared the prevalence of vitamin D sufficiency (25-hydroxy vitamin D (25(OH)D) concentration of ≥50 nmol/L), 25(OH)D concentration and the frequency of consuming vitamin D-rich foods among children aged 6–18 years-old using data from the nationally representative 2007/2009 and 2012/2013 Canadian Health Measures Surveys. Associations of sociodemographic, anthropometric, seasonal, and regional variables with achieving vitamin D sufficiency, 25(OH)D concentration, and consumption of vitamin D-rich foods were assessed using multiple logistic and linear regression models. 79% and 68% of children in 2007/2009 and 2012/2013 respectively, were vitamin D sufficient. The main dietary source of vitamin D was milk. Between 2007/2009 and 2012/2013, the frequency of milk and fish consumption declined, but egg and red meat consumption was unchanged. Age, income, weight status, season and ethnicity were associated with 25(OH)D concentration and vitamin D sufficiency. Vitamin D status declined after the upward revision of dietary guidelines for vitamin D, consequently, dietary intake was inadequate to meet sufficiency. Public health initiatives to promote vitamin D-rich foods and supplementation for Canadian children are needed.

## 1. Introduction

Vitamin D is an essential micronutrient for optimum health. Chronic insufficient intake may result in compromised health even in the absence of clinical signs of deficiency, resulting in “hidden hunger” that occurs when the quality of food eaten does not meet nutrient requirements [1]. Cutaneous synthesis through sun exposure is the main source of vitamin D_3_ [1,2], thus dietary vitamin D (vitamin D_2_ or vitamin D_3_) intake is critical to maintain proper vitamin D status among those who have limited sun exposure. Canada’s high latitude prevents year-round vitamin D synthesis through sun exposure [2,3]. Cutaneous synthesis of vitamin D is further limited during summer months due to public health recommendations to cover the arms and legs with clothing, use sunscreen, and avoid prolonged sun exposure [4] as preventive measures against skin cancer [4,5]. Inadequate sun exposure, together with the limited availability of vitamin D-rich foods [2,6] and poor food choices [7,8,9] presumably increase the risk of poor vitamin D status among Canadian children, along with a low prevalence of vitamin D supplement use [10,11,12]. 

To ensure optimal vitamin D status among Canadians and Americans, the dietary guidelines for vitamin D were revised in 2010 with the report by the Institute of Medicine released to the public in 2011 [13]. In 2010, the dietary intake of vitamin D for individuals aged 1–70 years was increased from an Adequate Intake of 200 IU to 600 IU as the Recommended Daily Allowance (RDA), which is the value presumed to meet or exceed the vitamin D requirement of 97.5% of the healthy population. At the same time, the Estimated Average Requirement (EAR) was established at 400 IU, which is the value that is estimated to meet the requirements of half of the population. The thresholds for dietary guidelines were set with the assumption of no or minimal sun exposure [13] and therefore it is expected that children will meet the guidelines through diet and supplementation alone. Serum or plasma 25-hydroxy vitamin D (25(OH)D) concentration is the established biomarker for vitamin D status [14]. The cut-off of 50 nmol/L is considered evidence of “sufficient” vitamin D concentration for the achievement and maintenance of bone health and as an indicator of achieving the RDA of 600 IU [13]. Despite the limited availability of natural dietary sources of vitamin D in Canada [6], only 2 food sources are mandatorily fortified with vitamin D: liquid cow’s milk with 35–40 IU/100 mL and margarine with at least 530 IU/100 g [15]. Following the revised dietary reference intakes for vitamin D, Health Canada recommended a daily vitamin D supplement of 400 IU for breastfed, healthy term infants and adults above the age of 50 years [6]. Health Canada also advised Canadians to follow *Canada’s Food Guide to Healthy Eating* which recommends that all Canadians older than 2 years consume 500 mL (2 cups) of vitamin-D fortified milk or soy beverages on a daily basis [16]. *Canada’s Food Guide to Healthy Eating* was last updated in 2007 and it is currently being revised.

Notwithstanding the current actions to promote vitamin D intake, vitamin D deficiency and insufficiency are still persistent among children living in Canada [10,11,17] and vitamin D intake from the diet is generally low [10,18]. However, no study has been conducted to determine whether vitamin D status has improved as per the intention of the revised 2010 dietary guidelines. Therefore, the present study aimed to identify and compare serum 25(OH)D concentrations, the frequency of consuming vitamin D-rich dietary sources and the prevalence of vitamin D sufficiency among Canadian children before and after the revision of the dietary guidelines. A second aim was to examine the sociodemographic, seasonal, geographical and anthropometric factors associated with serum 25(OH)D concentrations, consumption of vitamin D-rich foods, and the prevalence of vitamin D sufficiency. This assessment of vitamin D status and the intake of vitamin D-rich foods, following the 2010 revision of dietary guidelines to consume more vitamin D, will enable public health professionals, dieticians and policy makers to identify potential gaps in current vitamin D recommendations to ensure optimal vitamin D status among Canadian children.

## 2. Participants and Methods

### 2.1. Survey, Sample Design and Participants

The present study analyzed data collected from Cycle 1 (2007–2009) and Cycle 3 (2012–2013) of the Canadian Health Measure Survey (CHMS), which is a national comprehensive direct health measures survey representing Statistics Canada’s standard regional boundaries: Atlantic Provinces (Nova Scotia, New Brunswick, Newfoundland and Labrador, Prince Edward Island), Quebec, Ontario, the Prairie Provinces (Alberta, Manitoba and Saskatchewan) and British Columbia. Individuals aged 6–79 years-old participated in Cycle 1 and individuals aged 3–79 years-old participated in Cycle 3. Both Cycles included an in-home interview to gather demographic and health information, followed by the respondent’s visit to a Mobile Examination Centre for direct physical measurements (that is, height and weight) and biological specimen collection. All data were collected after receiving the participant’s consent and a parent or guardian provided consent on behalf of children below 14 years-old after receiving the child’s assent to participate. A parent or a guardian answered the questions with the assistance of the child if the participant was below 12 years-old. The Statistics Canada website (http://www.statcan.gc.ca) provides additional information on the CHMS. 

A multi-stage sampling design on the CHMS provided reliable estimates at the national level for age groups and gender. For Cycle 1, 15 sites were randomly selected from a total of 257 eligible sites that represented the Canadian population, east to west, with larger and smaller population densities using a systematic sampling method with probability proportional to each site’s population size. Similarly, 16 out of the 360 eligible collection sites were selected in Cycle 3. Dwellings were selected through random sampling from collection sites followed by stratified sampling of inhabitants within the dwellings based on age-groups [19,20,21]. Each cycle in the CHMS excluded about 4% of the population that were residents living in certain remote regions or on First Nations reserves and Aboriginal settlements, full-time members of the Canadian Forces, or the institutionalized population [19,20,21]. A detailed description of CHMS sampling framework can be found elsewhere [19,20,21]. 

Out of children aged 6–18 years who participated in CHMS Cycle 1 and Cycle 3, those who were pregnant or had an insufficient quantity of blood drawn were excluded. Consequently, data from 1740 and 1800 children from Cycle 1 and Cycle 3, respectively, were considered in the present study. 

### 2.2. Serum 25(OH)D Measurement

Serum 25(OH)D concentration was determined using the LIAISON^®^ 25-hydroxyvitamin D TOTAL assay on the Diasorin Liaison autoimmunoanalyzer (Diasorin, Ltd., Stillwater, Minnesota) using chemiluminescence immunoassay technology with the analytical detection limit of 10–375 nmol/L. Serum 25(OH)D analysis is further described in the *Vitamin D Reference Laboratory Standard Operating Procedures Manual* [22]. Between-run coefficients of variation for the assays in Cycle 1 and Cycle 3 were 12.7% and 13.0%, respectively. CHMS reference laboratory precision targets for <20 nmol/L, 20–100 nmol/L and >100 nmol/L were 15%, 10% and 12%, respectively. Meeting vitamin D sufficiency was defined as having at least 50 nmol/L of 25(OH)D in the circulation, deficiency as having 25(OH)D concentration <30 nmol/L, and inadequacy (insufficiency) as 30–50 nmol/L [13]. Based on the IOM report the cut-off for the “potential risk of adverse effects” was considered 25(OH)D concentrations >125 nmol/L.

### 2.3. Vitamin D-Rich Foods

Qualitative food frequency data were collected as part of the household questionnaire. Food frequency data on margarine consumption were not collected in CHMS Cycle 1. Therefore, only the information on the frequency of consumption of fish (excluding shell fish), cow’s milk (milk or flavored milk beverages or milk used in cereal), eggs (egg or egg dishes excluding egg dishes made with only egg whites), red meat (beef, hamburger, pork or lamb), and liver (including all types of liver such as beef, veal, pork or chicken but excluding liverwurst and liver pâté) within the previous month was included in the present study.

### 2.4. Other Covariates

Gender, age, household income, body weight status, ethnicity, and season were considered covariates of vitamin D sufficiency and 25(OH)D concentration. Vitamin D-containing supplement use was not considered in the present study because of restrictions put in place by Statistics Canada that prevented us from acquiring this data from Cycle 1. Household income was categorized as ≤50,000 CAD, >50,000–100,000 CAD or >100,000 CAD per year. Missing income data was imputed by Statistics Canada based on procedures found in the CHMS *Data User Guide: Cycle 1* [21] and CHMS *Data User Guide: Cycle 3* [20]. Standing height was measured to the nearest 0.01 mm using a fixed stadiometer (Quickmedical 235A, Issaquah, WA, USA) and body weight to the nearest 0.01 kg using a digital scale (Mettler Toledo 2256 VLC, Columbus, OH, USA). Body weight status was defined as “underweight,” “normal weight,” “overweight,” and “obese” using the Body Mass Index (BMI; weight/height^2^ in kg/m^2^) based on the WHO classification for children and adolescents [23]. “Underweight” and “normal weight” categories were combined into a single category due to the small proportion of underweight children. Ethnicity was dichotomized as “white” and “non-white” (i.e., Chinese, South Asian, Black, Filipino, Latin American, Southeast Asian, Arab, West Asian, Japanese, Korean, Aboriginal, and other ethnic backgrounds) based on variations in skin colour that determine the capacity to cutaneously synthesize vitamin D [17]. Season was defined as “winter” (December of the previous year, January, February), “spring” (March, April, May), “summer” (June, July, August) and “fall” (September, October, November) based on the date of the visit to Mobile Examination Centre to provide blood samples. 

### 2.5. Statistical Analyses

In order to accommodate the complex sampling design, all analyses were weighted to represent national estimates of individuals aged 6–18 years-old. Descriptive statistics were presented as means with bootstrap standard errors or percentages. The chi-square test was employed to compare the basic characteristics between the 2 Cycles—i.e., before and after the revision of dietary guidelines. Multiple logistic regression identified whether meeting vitamin D sufficiency among children had changed between Cycle 1—i.e., before the upward revision of dietary intake guidelines for vitamin D—and Cycle 3—i.e., following the revision of guidelines—and adjusted for gender, age, household income, body weight status, ethnicity, season, and geographical region. Multiple linear regression was employed to investigate the association of serum 25(OH)D concentration with these covariates. Dietary factors were not included as covariates in both logistic and linear regression models as they are in the causal pathway of the association between the revised dietary guidelines and achieving vitamin D sufficiency and 25(OH)D concentration. Milk, fish, egg and red meat consumption were each grouped into 2 frequency consumption categories and the covariates associated with their consumption before and after the revision of guidelines were identified in separate logistic regression models, after adjusting for other covariates. Liver was not included in regression analysis due to its low frequency in the diet. All analyses were carried out using Stata version 14.0 (Stata Corp, College Station, TX, USA) with statistical significance set at *p* < 0.05. The Health Research Ethics Board of the University of Alberta and Statistics Canada approved this study. All processes of CHMS were reviewed and approved by Health Canada and the Public Health Agency of Canada Research Ethics Board.

## 3. Results

The average age of participating children was 12.5 (bootstrap SE = 0.08) years in CHMS Cycle 1—i.e., 2007/2009, before the upward revision of dietary guidelines—and 12.2 (bootstrap SE = 0.08) years in CHMS Cycle 3—i.e., 2012/2013, following the revision of dietary guidelines. The season in which vitamin D status was assessed was similar in both CHMS Cycles, as were the sociodemographic, ethnic and anthropometric characteristics of children. There were differing proportions of children in the 6–9 years-old and 10–13 years-old age groups between the two Cycles (see Table 1). The most commonly consumed vitamin D-rich food was milk in both cycles—however, more children (80.2%) reported drinking milk once a day or more frequently in 2007/2009 compared with 2012/2013 (74.7%; *p* = 0.01). The prevalence of consuming fish more than once a week was higher in 2007/2009 (37.9%) compared with 2012/2013 (14.6%; *p* < 0.01). The frequency of red meat, egg and liver consumption before and after the revision of the dietary guidelines did not change significantly (see Table 1).

The mean 25(OH)D concentration of Canadian children in 2007–2009 was 71.0 nmol/L (95% CI = 67.0, 74.9; bootstrap SE = 1.9), which was significantly higher than the 60.8 nmol/L (95% CI = 54.1, 67.5; bootstrap SE = 3.2) measured in 2012–2013. Consequently, the proportion of children who were vitamin D sufficient was significantly higher before the revision of dietary guidelines than it was after—79.4% vs. 68.3%, respectively (*p* = 0.04). The prevalence of vitamin D deficiency (Cycle 1 = 4.3%; Cycle 3 = 6.5%) and insufficiency (Cycle 1 = 16.3%; Cycle 3 = 25.2%) both increased from Cycle 1 to Cycle 3. Only 2.8% and <1.0% of children in Cycle 1 and Cycle 3, respectively, had 25(OH)D concentrations >125 nmol/L, which is the cut-off for the “potential risk of adverse effects.”

Table 2 compares serum 25(OH)D concentration and achieving vitamin D sufficiency before and after the revisions of the dietary guidelines, while considering differences with respect to sociodemographic, anthropometric, geographical and seasonal factors. This revealed that children in 2012/2013 were 0.5 times as likely to achieve vitamin D sufficiency and had on average a 9.1 nmol/L lower serum 25(OH)D concentration relative to children in 2007/2009. Table 2 further shows that older children compared with younger children, children from low income families compared with high income families, overweight and obese children compared with under/normal-weight children, and children of non-white ethnicity compared with those of white ethnicity, were less likely to be vitamin D sufficient. Increasing age, non-white ethnicity and being overweight or obese were negatively associated with circulating 25(OH)D concentration, whereas household income was positively associated. Children were more likely to achieve sufficiency and to have higher 25(OH)D concentrations during summer and fall compared to winter months.

Table 3 shows whether the revision of dietary guidelines was associated with the consumption of vitamin D-rich dietary sources as well as the association of covariates with consumption of each dietary source. The consumption of cows’ milk on a daily basis or more frequently (OR = 0.7; 95% CI = 0.5, 1.0) and fish consumption more than once a week (OR = 0.2; 95% CI = 0.1, 0.4) were decreased from Cycle 1 to Cycle 3 when adjusted for potential confounders. Additionally, increasing age was associated with less frequent milk consumption, whereas non-white ethnicity was associated with more frequent fish and egg consumption but less frequent red meat consumption. Boys were more likely to frequently consume milk and red meat than girls.

## 4. Discussion

The present study used Canadian data collected from two nationally representative samples of 6–18 years-old children in 2007/2009 (CHMS Cycle 1) and 2012/2013 (CHMS Cycle 3). Children’s 25(OH)D concentrations declined between cycles despite revisions of the dietary guidelines in 2010 to increase intake of vitamin D. Consequently, the prevalence of vitamin D sufficiency decreased by 11.1% and the prevalence of deficiency increased by 2.2% from Cycle 1 to Cycle 3. The dietary guidelines presume that vitamin D will be derived from the diet, not from cutaneous synthesis through sun exposure. For Canadian children, natural sources of vitamin D are red meat, liver, fatty fish and egg yolk; although the main dietary source of vitamin D is milk mandatorily fortified with vitamin D [10,11]. We found that despite recommendations to increase dietary intake of vitamin D the frequency of fish and milk consumption declined among children after the revision of dietary guidelines and there was no increased consumption of other vitamin D containing foods. Regardless of the recommendation in Canada for children to consume milk on a daily basis [16], one fourth of children in our study failed to achieve this target following the revision of dietary guidelines in 2010 hence promotion of milk consumption seems critical for children to help them achieve vitamin D sufficiency [24]. Although the cutaneous synthesis of vitamin D during winter months is limited, the frequency of consuming vitamin D-rich dietary sources was not associated with seasonality.

Unlike American adolescents [25], Canadian children in our study did not show ethnic disparities in milk consumption, but differences were identified in the frequency of consumption of other vitamin D rich dietary sources. White children were less likely to consume fish and eggs, but more likely to consume red meat than non-white children which contrasts with the situation in the US where African-American adolescents have higher meat consumption than white children [25]. Red meat consumption was also lower among girls compared to boys as well as higher among children residing in Quebec than the Atlantic region.

Although it is essential to find efficient ways to improve vitamin D status, dietary intake alone may not be the best strategy considering our finding of low milk intake among some children and the evidence that obese children have higher requirements for vitamin D [2,26,27], lactose-intolerance among some ethnic groups [2,28], veganism among some members of the population [2], the limited number of commonly consumed natural [2,18,29] and fortified [15] dietary sources of vitamin D, and limited availability and accessibility to such foods [30,31,32]. Thus, dietary supplements could play a major role in meeting sufficient 25(OH)D concentrations [12,33], especially for those who live in Northern latitudes [34,35]. Despite this, only a small proportion of Canadian children take vitamin D supplements [10,11] and supplement use follows a J-shaped curve, with the highest prevalence of usage at either end of the age spectrum [12,13,14,15,16,17,18,19,20,21,22,23,24,25,26,27,28,29,30,31,32,33]. In Canada, supplementation is only recommended for infants and individuals over 50 years of age, which might explain why so few children take supplements [6]. 

The current study has limitations. We could not calculate vitamin D intake from the diet as information on the amounts of food consumed was not collected. Also, information on margarine consumption—which is mandatorily fortified with vitamin D in Canada—was not collected in Cycle 1. However, the contribution of margarine to vitamin D sufficiency is reported to be low for children [10,18]. We also could not include information about vitamin D supplementation into our analysis due to the inaccessibility of complete data in Cycle 1. For Cycle 3 we previously reported that 9.2% of children use vitamin D containing supplements and/or analogues [10]. Our study has many strengths. The CHMS followed quality control measures to maintain data quality, including interviews and biological specimen collection and analysis. Complete and accurate data were ensured by performing data validation halfway through and at the end of data collection. CHMS interview and laboratory data were comparable with other CHMS cycles, the Canadian Community Health Survey, and the US National Health and Nutrition Examination Survey to ensure that the data were consistent among those different data sources [20].

In conclusion, vitamin D insufficiency among Canadian children is on the rise despite upward revisions of dietary guidelines to improve vitamin D status. Given our findings, dietary intake may not be effective as a sole strategy for achieving vitamin D sufficiency in children. Public health initiatives to expand food-based strategies [18,29,36] together with establishing formal recommendations for vitamin D supplementation are merited as strategies to bridge the gap between current intake and the recommended threshold. Further studies are warranted that address knowledge and practices on dietary guidelines, and to ensure compliance with vitamin D deficiency/insufficiency prevention strategies. It is necessary in future studies to also identify the reasons why vitamin D status and the frequency of milk and fish consumption declined among Canadian children despite IOM recommendations to increase vitamin D intake through food. Possible reasons are (1) inadequate dissemination of information about the revised dietary guidelines or (2) confusion created by having two DRI cut-offs, i.e., RDA (600 IU/day) as the target at the individual or clinical level, and EAR (400 IU/day) at the population level [29,37]; or (3) criticisms suggesting that the revised DRI threshold [38,39,40,41,42] of 600 IU/day of intake may not be sufficient to achieve 25(OH)D concentration of 50 nmol/L; or (4) inability to meet DRI through the diet alone [11,12,13,14,15,16,17,18,19,20,21,22,23,24,25,26,27,28,29,30,31,32,33,34,35,36,37,38,39,40,41,42,43]; or (5) rise in the prevalence of obesity due to children’s lifestyles development toward indoor sedentary activities [44]; or (6) a combination of the five. Moreover, given the suboptimal vitamin D status of many Canadian children, health practitioners should be advised to probe caregivers about their children’s use of vitamin D supplements and consumption of vitamin-D rich foods. In summary, our results indicate that revising dietary guidelines alone is insufficient to improve vitamin D status and likely requires additional public health actions to promote vitamin D nutrition for children.

## Figures and Tables

**Table 1:** General characteristics of Canadian children, age 6 to 18 years, participating in the 2007/2009 (Cycle 1) and 2012/2013 (Cycle 3) Canadian Health Measures Surveys.

	Weighted Percentage of Children		*p*-Value ^‡^
	Cycle 1 (*n* = 1740)	Cycle 3 (*n* = 1800)	
***Demographic, Economic, Geographical, Anthropometric and Seasonal Factors***			
Age			
6–9 years	24.8	29.9	0.04
10–13 years	32.4	27.8	
14–18 years	42.8	42.3	
Gender			
Boys	51.6	51.9	0.79
Girls	48.4	48.1	
Household income			
<50,000 CAD	26.9	27.7	0.62
50,000–<100,000 CAD	40.3	36.3	
≥100,000 CAD	32.8	36.0	
Region of residence			
Atlantic	6.4	6.3	0.90
Quebec	21.6	21.6	
Ontario	39.0	40.3	
The Prairies	19.3	19.3	
British Columbia	13.7	12.5	
Ethnicity			
White	75.5	64.3	0.17
Non-white	24.5	35.7	
Body weight status			
Under weight	3.8	5.2	0.66
Normal weight	69.5	68.5	
Overweight	13.4	13.9	
Obese	13.3	12.3	
Season			
Winter	22.4	21.2	1.00
Spring	28.4	30.2	
Summer	25.4	25.0	
Fall	23.8	23.6	
***Dietary Factors***			
Milk			
Less than daily	19.8	25.3	0.01
Daily or more frequently	80.2	74.7	
Fish			
Once a week or less	62.1	85.4	<0.01
More than once a week	37.9	14.6	
Egg			
Once a week or less	27.6	28.3	0.77
More than once a week	72.4	71.7	
Red meat			
Once a week or less	8.7	13.2	0.13
More than once a week	91.3	86.8	
Liver			
Once a week or less	98.4	98.9	0.40
More than once a week	1.6	1.1	

^‡^ Chi-sq test that compares each characteristic between two cycles.

**Table 2:** Associations of the revision of dietary guidelines and demographic, economic, geographical, anthropometric and seasonal factors with the likelihood of achieving vitamin D sufficiency and with serum 25(OH)D concentrations among Canadian children aged 6 to 18 years, participating in the 2007/2009 (Cycle 1) and 2012/2013 (Cycle 3) Canadian Health Measures Surveys.

Covariate	Achieving Vitamin D Sufficiency ^‡,†^		Serum 25(OH)D Concentration ^‡,†^	
	OR (95% CI)	*p* Value	β (95% CI)	*p* Value
Intercept			69.3 (55.8, 82.8)	<0.01
Cycle #				
Cycle 1	1.0		0.0	
Cycle 3	0.5 (0.3,0.9)	0.02	−9.1 (−14.4, −3.7)	<0.01
Age				
6–9 years	1.0		0.0	
10–13 years	0.5 (0.3, 0.7)	<0.01	−6.9 (−8.8, −4.9)	<0.01
14–18 years	0.3 (0.2, 0.5)	<0.01	−8.8 (−12.3, −5.2)	<0.01
Gender				
Boys	1.0		0.0	
Girls	1.0 (0.7, 1.3)	0.81	0.4 (−1.9, 2.8)	0.70
Household income				
<50,000 CAD	1.0		0.0	
50,000–<100,000 CAD	1.6 (1.2, 2.2)	<0.01	4.4 (1.7, 7.0)	<0.01
≥100,000 CAD	2.1 (1.4, 3.2)	<0.01	7.5 (4.9, 10.1)	<0.01
Region of residence				
Atlantic	1.0		0.0	
Quebec	0.5 (0.2, 1.4)	0.22	−4.0 (−16.5, 8.4)	0.51
Ontario	1.1 (0.4, 2.7)	0.89	0.3 (−11.5, 12.1)	0.96
The Prairies	1.0 (0.2, 4.2)	0.97	−1.6 (−19.3, 16.0)	0.85
British Columbia	0.9 (0.3, 2.4)	0.79	−0.4 (−12.9, 12.2)	0.95
Ethnicity				
White	1.0		0.0	
Non-white	0.2 (0.2, 0.4)	<0.01	−14.0 (−18.1, −10.0)	<0.01
Body weight status				
Under/Normal weight	1.0		0.0	
Overweight	0.6 (0.4, 0.8)	0.01	−3.6 (−6.8, −0.3)	0.03
Obese	0.4 (0.2, 0.6)	<0.01	−8.6 (−10.9, −6.2)	<0.01
Season				
Winter	1.0		0.0	
Spring	1.7 (0.7, 3.7)	0.19	4.8 (−2.8, 12.4)	0.21
Summer	5.1 (2.2, 11.9)	<0.01	17.9 (10.1, 25.6)	<0.01
Fall	4.1 (1.8, 9.2)	<0.01	14.4 (4.9, 24.0)	<0.01

Abbreviations: OR, Odds Ratio; β, co-efficient; CI, Confidence interval. ^‡^ Results of aged 6–18 years old children participated in CHMS cycles 1 and 3 were weighted to represent national estimates and adjusted for all covariates in the table. ^†^ Adjusted for all other covariates in the table.

**Table 3:** Associations of the revision of dietary guidelines and demographic, economic, geographical, anthropometric and seasonal factors with the frequency of consuming vitamin D-rich foods among Canadian children aged 6 to 18 years, participating in the 2007/2009 (Cycle 1) and 2012/2013 (Cycle 3) Canadian Health Measures Surveys.

Covariate	Consuming Milk Daily or More Frequently ^‡,†^		Consuming Fish Weekly or More Frequently ^‡,†^		Consuming Egg Weekly or More Frequently ^‡,†^		Consuming Red Meat Weekly or More Frequently ^‡,†^	
	OR (95% CI)	*p* Value	OR (95% CI)	*p* Value	OR (95% CI)	*p* Value	OR (95% CI)	*p* Value
Cycle #								
Cycle 1	1.0		1.0		1.0		1.0	
Cycle 3	0.7 (0.5, 1.0)	0.05	0.2 (0.1, 0.4)	<0.01	0.9 (0.8, 3.1)	0.54	0.7 (0.3, 1.3)	0.22
Age								
6–9 years	1.0		1.0		1.0		1.0	
10–13 years	0.5 (0.3, 0.7)	<0.01	1.0 (0.8, 1.3)	0.96	0.9 (0.7, 1.2)	0.64	1.0 (0.7, 1.5)	0.97
14–18 years	0.2 (0.2, 0.3)	<0.01	0.9 (0.7, 1.1)	0.28	1.0 (0.7, 1.5)	0.84	1.0 (0.6, 1.6)	0.92
Gender								
Boys	1.0		1.0		1.0		1.0	
Girls	0.6 (0.5, 0.8)	<0.01	0.9 (0.7, 1.2)	0.54	0.8 (0.6, 1.0)	0.11	0.5 (0.3, 0.7)	<0.01
Household income								
<50,000 CAD	1.0		1.0		1.0		1.0	
50,000–<100,000 CAD	1.0 (0.7, 1.3)	0.85	1.0 (0.7, 1.4)	0.99	1.1 (0.8, 1.6)	0.46	1.1 (0.7, 1.8)	0.57
≥100,000 CAD	1.1 (0.8, 1.5)	0.63	1.3 (1.0, 1.9)	0.07	1.1 (0.7, 1.5)	0.73	1.1 (0.6, 2.1)	0.74
Region of residence								
Atlantic	1.0		1.0		1.0		1.0	
Quebec	1.0 (0.4, 2.7)	0.95	2.0 (0.5, 7.9)	0.29	1.3 (0.7, 2.2)	0.33	4.2 (1.2, 14.3)	0.02
Ontario	1.1 (0.4, 2.8)	0.84	1.2 (0.3, 4.8)	0.78	1.1 (0.7, 1.9)	0.65	1.2 (0.3, 4.5)	0.76
The Prairies	1.2 (0.5, 2.9)	0.73	1.3 (0.3, 6.3)	0.71	1.2 (0.7, 2.2)	0.41	1.5 (0.2, 8.6)	0.66
British Columbia	0.9 (0.3, 2.4)	0.81	1.4 (0.2, 9.0)	0.71	1.4 (0.7, 2.6)	0.30	1.3 (0.2, 6.8)	0.74
Ethnicity								
White	1.0		1.0		1.0		1.0	
Non-white	0.8 (0.5, 1.1)	0.16	1.9 (1.2, 3.0)	<0.01	1.6 (1.0, 2.5)	0.05	0.4 (0.2, 0.9)	0.03
Body weight status								
Under/Normal weight	1.0		1.0		1.0		1.0	
Overweight	0.9 (0.6, 1.2)	0.48	1.3 (0.9, 1.9)	0.14	1.1 (0.8, 1.4)	0.55	1.0 (0.5, 1.8)	0.99
Obese	1.1 (0.7, 1.7)	0.67	0.7 (0.4, 1.0)	0.08	0.8 (0.6, 1.2)	0.40	0.9 (0.6, 1.5)	0.69
Season								
Winter	1.0		1.0		1.0		1.0	
Spring	1.0 (0.6, 1.8)	0.94	0.8 (0.4, 1.7)	0.59	1.4 (0.8, 2.4)	0.23	1.4 (0.5, 4.2)	0.49
Summer	1.1 (0.6, 2.0)	0.83	0.9 (0.5, 1.7)	0.70	1.5 (0.8, 2.6)	0.18	1.2 (0.4, 3.2)	0.75
Fall	1.3 (0.7, 2.7)	0.38	1.0 (0.5, 1.9)	1.00	1.2 (0.7, 2.0)	0.41	2.0 (0.6, 6.3)	0.22

Abbreviations: OR, Odds Ratio; CI, Confidence interval. ^‡^ Results of aged 6–18 years old children participated in CHMS cycles 1 and 3 were weighted to represent national estimates and adjusted for all covariates in the table. ^†^ Adjusted for all covariates in the table.
